# Proteolysis of ToxR is controlled by cysteine‐thiol redox state and bile salts in *Vibrio cholerae*

**DOI:** 10.1111/mmi.14125

**Published:** 2018-10-25

**Authors:** Mareike Lembke, Nina Pennetzdorfer, Sarah Tutz, Michael Koller, Dina Vorkapic, Jun Zhu, Stefan Schild, Joachim Reidl

**Affiliations:** ^1^ Institute of Molecular Biosciences University of Graz Humboldtstraße 50 Graz A‐8010 Austria; ^2^ Department of Microbiology University of Pennsylvania Philadelphia PA 19104‐6076 USA; ^3^ BioTechMed‐Graz Graz A‐8010 Austria

## Abstract

In *Vibrio cholerae*, virulence gene expression is regulated by a transmembrane‐localized transcription factor complex designated as ToxRS. ToxR harbours two cysteines in the periplasmic domain that can form inter‐ and intramolecular disulfide bonds. In this study, we investigated the σ^E^‐dependent inner membrane proteolysis of ToxR, which occurs via the periplasmic‐localized proteases DegS and DegP. Both proteases respond to the redox state of the two cysteine thiol groups of ToxR. Interestingly, in the presence of sodium deoxycholate, ToxR proteolysis is blocked independently of ToxS, whereas ToxR activation by bile salts requires ToxS function. From these data, we identified at least two levels of control for ToxR activation by sodiumdeoxycholate. First, bile inhibits ToxR degradation under starvation and alkaline pH or under conditions in which DegPS responds to the reduced disulfide bonds of ToxR. The second level links bile to ToxRS complex formation and further activation of its transcription factor activity. Overall, our data suggest a comprehensive bile sensory function for the ToxRS complex during host colonization.

## Introduction

Studies on *Vibrio cholerae* pathogenesis have revealed that the production of cholera toxin (CT) and toxin‐coregulated pili (TCP) is coordinated by a regulatory network that has been historically referred to as the ToxR regulon (Matson *et al.*, 2007). This system is comprised of several transcriptional factors, which include AphAB, TcpPH, ToxRS and ToxT (Miller and Mekalanos, 1984; Miller *et al.*, 1989; DiRita *et al.*, 1991; Hase and Mekalanos, 1998; Skorupski and Taylor, 1999). The AphAB complex, in which *aphA* is under the transcriptional control of the LuxOP quorum‐sensing system (Rutherford *et al.*, 2011), is active under anaerobic conditions (Kovacikova *et al.*, 2010), regulates *tcpPH* (Skorupski and Taylor, 1999) and enhances *toxRS* transcription (Xu *et al.*, 2010). TcpPH is a labile complex that is activated via bile salts, which cause rearrangements of TcpP‐intramolecular disulfide bonds to promote an intermolecular disulfide bond (Yang *et al.*, 2013). In contrast, under virulence‐promoting conditions, ToxR is constitutively expressed (Kanjilal *et al.*, 2010), and together with TcpP activates the transcription of *toxT* (Higgins and DiRita, 1994; Hase and Mekalanos, 1998; Bina *et al.*, 2003; Childers and Klose, 2007). ToxT subsequently activates the transcription of the *ctx* and *tcp* loci. *V. cholerae* strains that lack either TcpP, ToxT or ToxR do not produce CT or TCP and are non‐virulent (Champion *et al.*, 1997). ToxR directly regulates the transcription of many genes (Bina *et al.*, 2003), the best characterized of which are *ompT* and *ompU* (Miller and Mekalanos, 1988), which encode the major porins of *V. cholerae*.

Structurally, ToxR is related to the OmpR‐type regulators (Ottemann *et al.*, 1992). The N‐terminus of ToxR is located in the cytoplasm and contains a winged helix‐turn‐helix DNA‐binding motif, followed by a single transmembrane domain (TM) and a periplasmic C‐terminal domain (Miller *et al.*, 1987). Experimentally defined operator binding sites of ToxR are termed ToxR boxes and have been identified 40 to 180 bp upstream of the *ctx*, *ompU/T* and *toxT* promoters (Pfau and Taylor, 1996; Goss *et al.*, 2013). As demonstrated by domain analysis, the ToxR TM segment functions in ToxR activity and may be involved in a bile‐dependent ToxR activation (Dziejman *et al.*, 1999; Crawford *et al.*, 2003; Hung and Mekalanos, 2005). The ToxR periplasmic domain has been proposed to be a sensor for environmental stimuli, containing two cysteine residues at amino acid positions 236 and 293 that can form homodimer or intramolecular disulfide bonds (Ottemann and Mekalanos, 1996). Recently, we showed in greater detail that intramolecular disulfide bond formation in ToxR produces an active conformation of ToxR that is necessary for the proper regulation of the porin genes *ompU* and *ompT* but not for the activation of *toxT* transcription. Other transcriptional regulators also contain cysteine residues, including AphB and TcpP. Interestingly, the reduction of the cysteine thiol groups in some of these regulators has been shown to be important for virulence gene regulation and is dependent on oxygen limitation and the presence of bile salts (Liu *et al.*, 2011; Yang *et al.*, 2013). Additionally, cysteine residues in TcpP and TcpH have been reported to be crucial for protein stability (Morgan *et al.*, 2015), indicating that replacing these cysteine residues can disrupt inter‐ and intramolecular‐disulfide bonds within TcpP and TcpH and alter TcpPH stability.

Similar to *tcpH* and *tcpP*, a second gene is co‐transcribed with *toxR*, termed *toxS* (Miller *et al.*, 1989b). ToxS is also an inner membrane‐localized protein (DiRita *et al.*, 1991) with only 6‐8 amino acids exposed to the cytoplasm, followed by a single TM domain and a C‐terminal region located in the periplasm. The current model suggests that ToxR and ToxS are interacting partners in the periplasm (DiRita and Mekalanos, 1991) and form heterodimers (Ottemann and Mekalanos, 1996). The intramolecular disulfide form of ToxR monomers was previously shown to be a binding partner for ToxS and the inability to form intramolecular disulfide bonds is associated with the attenuated regulation of OmpT and OmpU porin expression (Fengler *et al.*, 2012). Furthermore, knockout mutations of *toxS* negatively influence the transcriptional activity of ToxR (Miller *et al.*, 1989), suggesting that either ToxS facilitates the activity of ToxR or that ToxS affects ToxR protein stability (DiRita and Mekalanos, 1991; Pfau and Taylor, 1998). Recently, it was demonstrated that ToxR is subject to proteolysis under starvation and alkaline pH conditions both in a classical and an El Tor O1 isolate (Almagro‐Moreno *et al.*, 2015a; 2015b). ToxS plays a crucial role in this process, since RseP‐ and site‐1‐mediated proteolysis can either be prevented by WT ToxS or promoted by mutant ToxS^L33S^ (Almagro‐Moreno *et al.*, 2015a; 2015b). Furthermore, the interaction between ToxR and ToxS has been described to be influenced by the binding of bile acids, which results in an enhanced active transcriptional complex, influencing OmpU and OmpT expression (Midgett *et al.*, 2017).

The recently published data addressing the ToxRS interaction with bile acids and ToxR proteolysis prompted us to elucidate the molecular relationship between ToxRS activation and stability. In this study, we describe two major routes of ToxR proteolysis. One route involves DegS and DegP, which respond towards the redox state of the ToxR cysteine residues. The other route appears to be independent of the redox state but is sensitive to starvation and alkaline conditions. However, both degradation paths are inhibited if bile salts (sodium deoxycholate) are present in the environment. Since bile salts control proteolysis and influence the activity of the ToxRS transcriptional complex, our data extend the current model of ToxRS and indicate a bile acid‐sensing function of ToxRS.

## Results

### ToxR protein stability depends on its redox state, its interaction with ToxS and the proteases DegS and DegP

**Figure 1:**
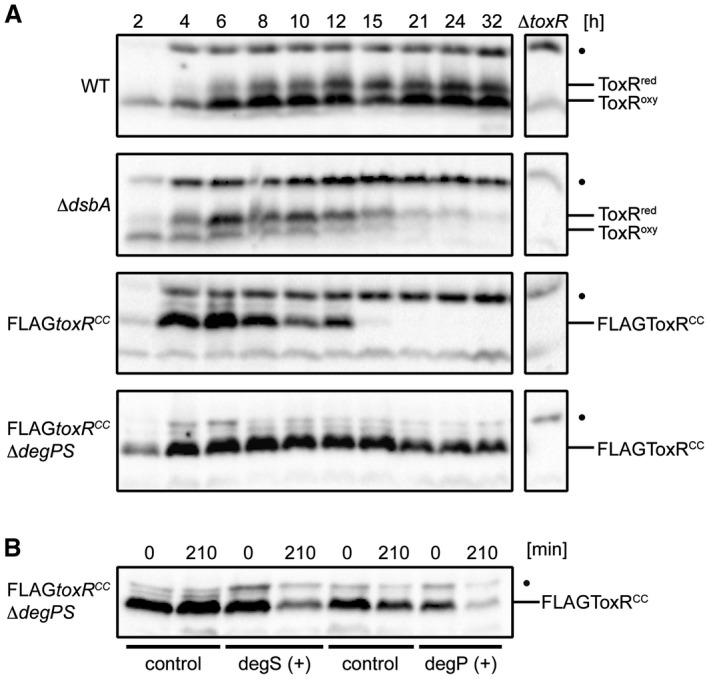
The cysteine oxidation and reduction state of ToxR affects protein stability in a DegPS‐dependent way. A. Shown are ToxR immunoblots of *V. cholerae* WT, *dsbA*, FLAG*toxR^CC^* and FLAG*toxR^CC^degPS* grown in M9 maltose. B. Complementation studies of *V. cholerae* FLAG*toxR^CC^degPS* are demonstrated by ToxR immunoblots. Cells harboured pBAD18‐KandegS, pMMB67EHdegP or the corresponding plasmid controls. Protein biosynthesis was inhibited by the addition of Cm. Immunoblots were performed under standard nonreducing Laemmli buffer conditions utilizing α‐ToxR antiserum. The migration patterns of ToxR^red/oxy^ are indicated. (•): Represents a nonspecific cross‐reacting background band.

As previously demonstrated, cysteine residues within the periplasmic domain of ToxR contribute to inter‐ and intramolecular disulfide bonds and have implications for ToxR transcription factor activity (Ottemann and Mekalanos, 1996; Fengler *et al.*, 2012). Prompted by the newly described regulated intramembrane proteolysis (RIP) of ToxR (Almagro‐Moreno *et al.*, 2015b), we revisited the cysteine replacement mutant *toxR^CC^*, which has a very low ToxR activation phenotype (Fengler *et al.*, 2012) and characterized ToxR^CC^ protein stability. For these experiments, we compared ToxR and ToxR^CC^ in WT, *dsbA*, FLAG*toxR^CC^*, FLAG*toxR^CC^degPS* (Fig. 1A), *dsbC* and *degSdsbA* *V. cholerae* strains (Fig. S1). From these cultures, whole cell lysates (WCL) were generated from cells grown in M9 maltose media for up to 32 h, solubilized in Laemmli buffer under nonreducing conditions and used for SDS‐PAGE followed by the immunoblot analysis. Two protein bands were observed, the reduced (ToxR^red^) and the oxidized (ToxR^oxy^) form, the latter of which contains an intramolecular disulfide bond (Fig. 1A) (Otteman and Mekalanos, 1996). At time points 6‐32 h, both, ToxR^red^ and ToxR^oxy^ were observed in the WT strain and no degradation was detected. In contrast, the endogenous ToxR in the *dsbA* strain and the FLAGToxR^CC^ mutant could hardly be detected past the 12 h time point (Fig. 1A), corresponding to an early stationary growth phase (Fig. S2). In the *dsbA* mutant, ToxR^red^ became the major protein form of monomeric ToxR from time point 6 h onwards. These results support the conclusion that DsbA acts as an oxido‐reductase on ToxR, forming intramolecular disulfide bonds and affecting ToxR stability. A *dsbC* mutant exhibited a stable ToxR expression profile with less recognizable ToxR^red^ when compared to the WT strain (Fig. S1). To assess DegS and DegP protease activities, we constructed *degSdsbA and *FLAG*toxR^CC^degPS *mutant strains and monitored ToxR and FLAGToxR^CC^ respectively. As observed in the *degSdsbA* mutant (Fig. S1), ToxR exhibited a prolonged and stable expression period, suggesting the participation of DegS during ToxR proteolysis. However, when compared to the WT strain, ToxR degradation was observed after incubating for 21‐24 h, indicating the presence of additional active proteases. As a result of these observations, we monitored a FLAG*toxR^CC^degPS *strain (Fig. 1A) and showed that ToxR^CC^ indeed stayed stable over 32 h if compared to the *FLAGtoxR^CC^* strain. To further elucidate the contribution of the two proteases DegS and DegP, the knockout mutant strain FLAG*toxR^CC^degPS *was used to perform complementation studies. The expression of either of these proteases in trans led to increased ToxR^CC^ degradation compared to the strains harbouring control plasmids (Fig. 1B). Furthermore, a *degS* knockout showed impaired σ^E^ response with significantly decreased *degP* transcription (Fig. S3). These data provide strong genetic evidence that the DegS and DegP proteases independently promote ToxR degradation if ToxR^red^ or FLAGToxR^CC^ is encountered.

**Figure 2:**
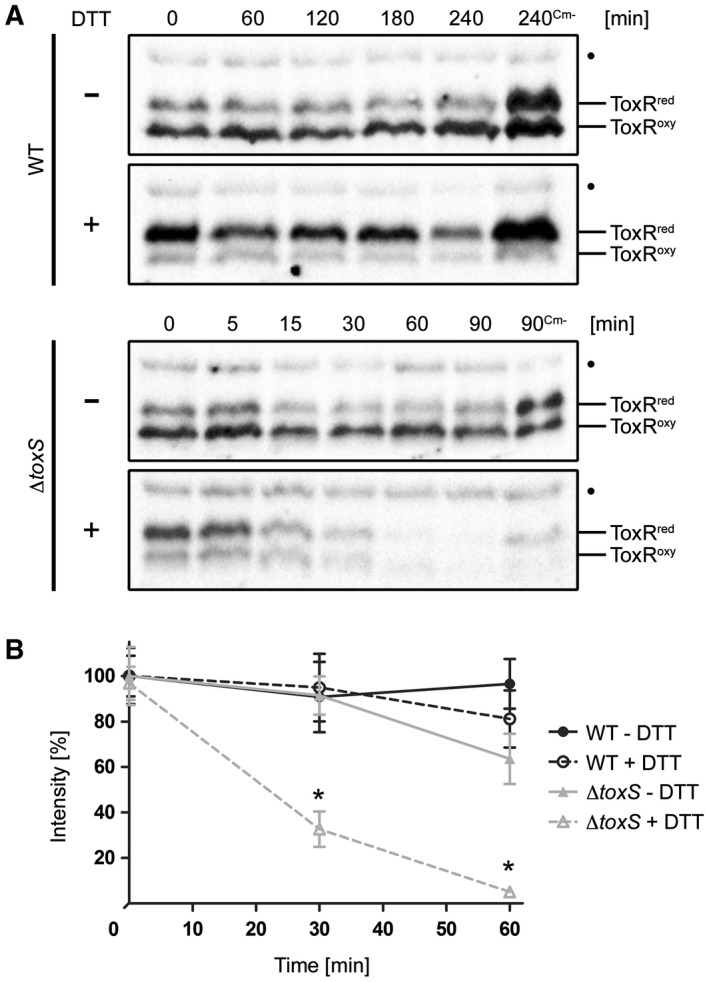
Effects of DTT treatment on the redox state and protein stability of ToxR in *V. cholerae* WT and *toxS* mutants. ToxR temporal stability levels were measured by the immunoblot analysis in WT and *toxS* mutant strains grown in M9 maltose with or without DTT (+/–). Protein biosynthesis was inhibited by the addition of Cm. Samples without chloramphenicol (Cm‐) served as negative controls. A. Immunoblots of WT and *toxS* WCL were performed under standard nonreducing Laemmli buffer conditions utilizing α‐ToxR antiserum. The migration patterns of ToxR^red/oxy^ are indicated. (•): Represents a nonspecific cross‐reacting background band. B. Graphs show band intensities (%) of WCL samples treated under reducing Laemmli buffer conditions defined by densitometry of similar blots (see one set of representative immunoblots Fig. S4). For each time point, the sample number was n ≥ 6 and the mean values with standard deviation are shown. Two‐way ANOVA with Bonferroni post hoc analysis indicates significant differences between *toxS* strains without (grey filled triangle, solid line) and *toxS* cells with DTT (grey open triangle, dotted line) with *P* < 0.001 at time points 30 and 60 min. No significant differences were seen between WT without DTT (black filled circle, solid line) and WT incubated with DTT (black open circle, dotted line).

To further characterize the instability of ToxR^red^ and the involvement of ToxS, temporal expression levels of chromosomally produced ToxR protein were examined in the WT and *toxS* mutant strains (Fig. 2A and B). To prevent de novo protein biosynthesis, chloramphenicol was added to monitor the fate of the ToxR protein. In this assay, cells were incubated with or without sublethal concentrations of the reducing agent DTT to promote the conversion of ToxR^oxy^ into ToxR^red^. Without the DTT treatment, the amount of ToxR^oxy^ was increased over ToxR^red^, while the opposite effect was detected in the presence of DTT in the WT and *toxS* strains. In these assays, ToxR was observed to remain stable in WT cells regardless of the presence or absence of DTT, whereas in the *toxS* mutant, increased ToxR degradation was detected in DTT‐treated cells (Fig. 2A). Using densitometric analysis, we determined that this result was significant (Fig. 2B, Fig. S4). Intriguingly, proteolysis was observed for ToxR^CC^ or for ToxR in a *dsbA* strain starting at time point 15 h (Fig. 1A). Therefore, we tested the WT and *toxS* knockout mutant strains grown to 15 h for ToxR stability (Fig. S5). After incubation, the cultures were divided and further incubated with or without DTT for an additional 6 h. Similar to the results presented in Fig. 2A, but without the addition of chloramphenicol, the greatly increased proteolysis of ToxR was only visible in the *toxS* knockout mutant in the presence of DTT (Fig. S5). Since DTT‐treated cells predominantly express ToxR^red^, we concluded that ToxR proteolysis correlates with a cysteine‐reduced state that is enhanced in the absence of ToxS. Importantly, we have no evidence for the selective degradation of a specific form of ToxR^red/oxy^. In addition, we obtained no evidence that DTT increases the proteolysis of ToxR^CC^ (Fig. S6); therefore, we conclude that DTT does not activate DegS and DegP. Furthermore, the involvement of DegS was confirmed in this assay, showing that in a *toxSdegS* double mutant, no ToxR degradation was observed, with or without DTT treatment (Fig. S7).

### Characterization of the DegS‐dependent ToxR and RseA proteolysis

**Figure 3:**
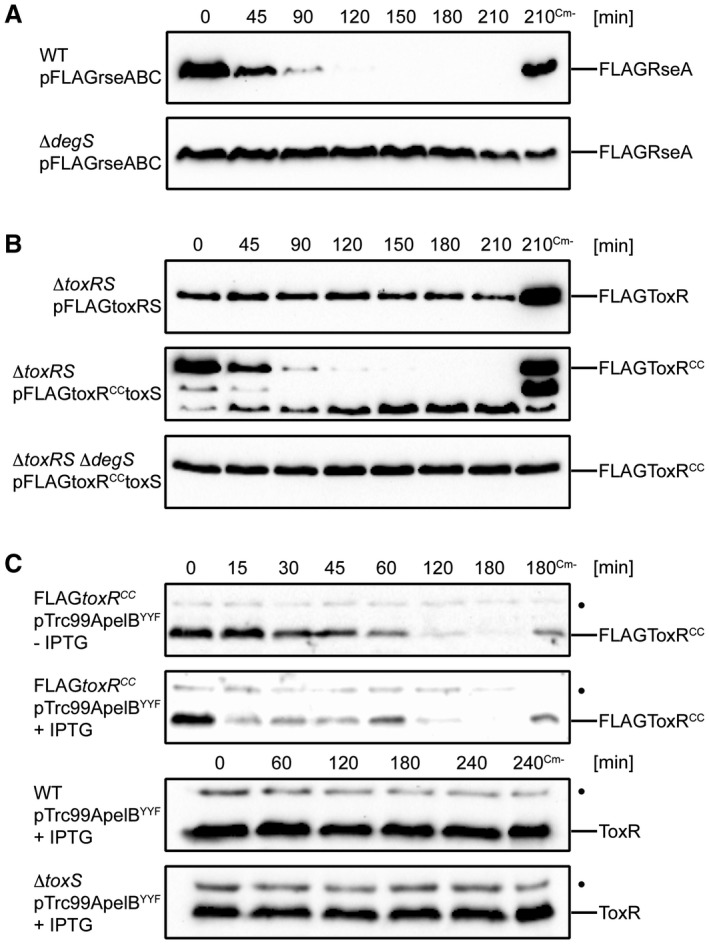
FLAGToxR^CC^ undergoes DegS‐regulated proteolysis, which can be enhanced upon the overexpression of a synthetic C‐terminal OmpU fragment (YYF). Degradation assay was conducted with plasmid‐carrying cells grown in M9 maltose to mid‐log growth phase. Cultures were induced with IPTG for 1 h followed by inhibition of protein translation by Cm. Samples without chloramphenicol (Cm‐) served as negative controls. A. Proteolysis of anti‐sigma factor RseA is controlled by site‐1 protease DegS. Immunoblots utilizing anti‐FLAG antibodies show temporal stability levels of FLAGRseA in WT and *degS* background. B. Regulated proteolysis of FLAGToxR^CC^ is controlled by DegS. Immunoblots utilizing anti‐FLAG antibodies are showing temporal stability levels of FLAGToxR^CC^ in *toxRS* and *toxRSdegS* background. C. DegS‐PDZ activation by a synthetic C‐terminal OmpU fragment (YYF) induces an acceleration of FLAGToxR^CC^ degradation. Immunoblots utilizing α‐ToxR antiserum show chromosomal expressed levels of ToxR or FLAGToxR^CC^. (•): Represents a nonspecific cross‐reacting background band.

DegS is a serine site‐1 protease and a stress‐sensor protein that activates the σ^E^ response pathway by recognizing unfolded OMPs in the periplasm of *Escherichia coli* (Lima *et al.*, 2013). Several studies of the σ^E^ pathway in *V. cholerae* have been published (Kovacikova and Skorupski, 2002; Ding *et al.*, 2004; Mathur *et al.*, 2007; Davis and Waldor, 2009) and RseP, DegS and DegP were recently shown to participate in ToxR proteolysis (Almagro‐Moreno *et al.*, 2015b). However, the release of σ^E^ via the activity of DegS was not well studied in these investigations. To evaluate DegS activity against its known substrate, the anti‐sigma factor RseA homologue of *V. cholerae* was monitored in a proteolysis assay (Fig. 3A). We observed that FLAGRseA was a substrate for the DegS protease under the conditions tested, as was FLAGToxR^CC^ monitored in *degS^+/–^* strains (Fig. 3B). For ToxR^CC^ several residual degradation products at approximately 31 and 26 kDa were observed, as proteolysis may have been incomplete due to its overexpression from a plasmid. Of note, the WT ToxR protein remained stable, even when overexpressed from a plasmid. Furthermore, as shown in Fig. 3C, the degradation of FLAGToxR^CC^ was accelerated in the presence of stressor molecules, which was accomplished via the over‐expression of a synthetic C‐terminal OmpU fragment derived from pTrc99ApelB^YYF^ (for a detailed description see Experimental Procedures). This assay was performed because it has been shown that the C‐terminal peptides derived from stress‐induced, unfolded porins act as binding partners for the PDZ domain of DegS, which in turn leads to σ^E^ pathway activation (Walsh *et al.*, 2003; Wilken *et al.*, 2004; Chaba *et al.*, 2007). In contrast, no activation of proteolysis was observed for the WT ToxR, neither in the absence nor in the presence of ToxS. Taken together, these results confirm that DegS acts proteolytically on the *V. cholerae* homologue of RseA, as shown in *E. coli*. In agreement with these observations, our data indicate that the activation of DegS triggered by C‐terminal YYF oligopeptide exposure also increases FLAGToxR^CC^ proteolysis.

### Bile salts interfere with ToxR proteolysis

**Figure 4:**
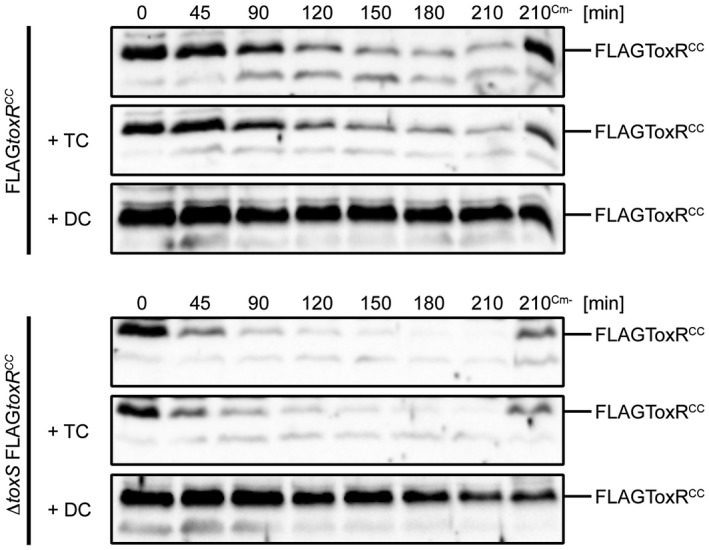
Sodium deoxycholate (DC) protects FLAGToxR^CC^ degradation in WT and in *toxS* mutant strains. Degradation assays of *V. cholerae* FLAG*toxR^CC^* and *toxS* FLAG*toxR^CC^* strains are subjected to immunoblotting using α‐ToxR antiserum. Cells were grown in M9 maltose in the absence or presence of 0.1% sodium taurocholate or sodium deoxycholate. Protein biosynthesis was inhibited by the addition of Cm, whereas samples without chloramphenicol (Cm‐) served as negative controls. Shown in Fig. S9 and S10 are Kang‐stained gels, which represent loading and quality controls to monitor influences of bile salts on protein expression patterns.

A well‐known effect of bile salts on *V. cholerae* is the activation of the ToxR transcriptional activity, which leads to high levels of *ompU* transcription and *ompT* repression (Provenzano *et al.*, 2000; Midgett *et al.*, 2017). Bile salts do not activate *toxRS* transcription per se (Mey *et al.*, 2015), but this surfactant may influence ToxRS interaction strength, as described recently (Midgett *et al.*, 2017). Therefore, we focused on the effects of bile salts on FLAGToxR^CC^ stability, with the results showing that DC, but not TC, could inhibit FLAGToxR^CC^ proteolysis (Fig. 4) in a ToxS‐independent manner. To determine whether DC inhibits DegS activity directly, a FLAGRseA degradation assay was performed. The results did not reveal an inhibition of DegS‐dependent proteolysis of FLAGRseA (Fig. S8), indicating that the DC‐mediated inhibition is ToxR specific but does not target the inhibition of the protease DegS. Interestingly, adjusted WCLs analysed by SDS‐PAGE and Kang staining revealed DC, but not TC, ToxR‐induced OmpU expression (Fig. S9 and S10).

**Figure 5:**
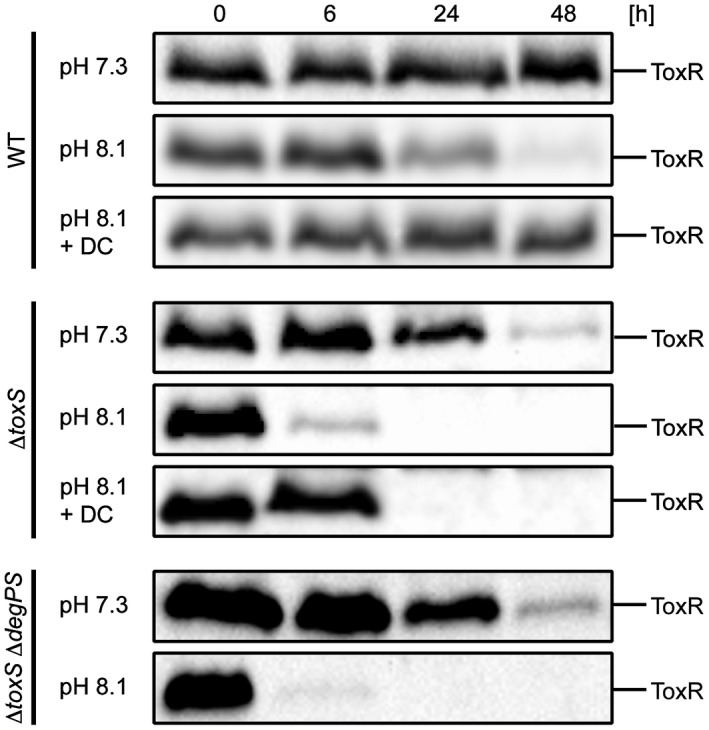
ToxR proteolysis under starvation and alkaline pH conditions is inhibited by DC. Shown are immunoblots of WCL samples of ToxR in WT, *toxS* and *toxSdegPS* strains treated under reducing Laemmli buffer conditions. Cells were grown in LB medium ON and then shifted into PBS (pH 7.3), alkaline PBS (pH 8.1) and alkaline PBS (pH 8.1) supplemented with DC (0.01%). Immunoblots were performed utilizing α‐ToxR antiserum.

As reported previously (Almagro‐Moreno *et al.*, 2015b), ToxR proteolysis was identified under conditions of starvation and alkaline pH incubation (PBS buffer pH 8.1). We detected ToxR in the WT strain and observed that DC also inhibits ToxR degradation under starvation and alkaline incubation conditions (Fig. 5). We also monitored ToxR stability in a *toxS* knockout mutant and monitored enhanced ToxR instability, which was partially stabilized by added DC. Additionally, we detected decreased ToxR levels under alkaline PBS conditions in the *toxSdegPS* triple mutant, indicating that other proteases recognize ToxR as a substrate.

**Figure 6:**
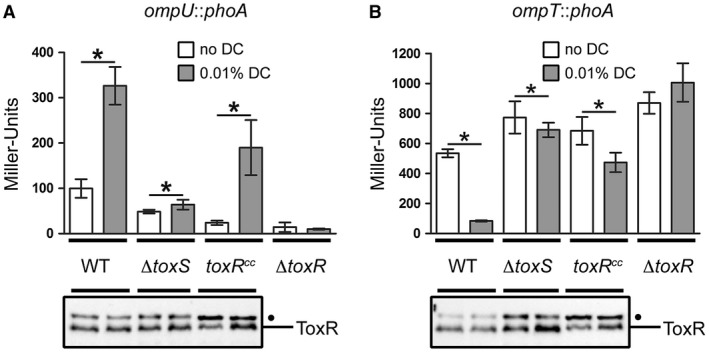
ToxR transcriptional control of *ompT* and *ompU* is dependent on ToxS under DC activating conditions. Shown are reporter gene activities of alkaline phosphatase PhoA (Miller Units) linked as operon fusions to either *ompU* (A) or *ompT* (B) in *V. cholerae* WT, *toxS*, FLAG*toxR^CC^* and *toxR* strains. Simultaneously, immunoblot analyses were performed utilizing α‐ToxR antiserum. Strains were grown in M9 maltose in the presence (dark bars) or absence (open bars) of 0.01% DC until OD_600_ of 0.8‐1. Data shown are mean and standard deviation of six independent samples for each condition. The asterisks indicate a statistically significant difference between bile salt‐treated and non‐treated cells, with *P*‐values by paired *t*‐test (*): *P* < 0.05. (•): Represents a nonspecific cross‐reacting background band.

As the interaction between bile salts and purified ToxR was published recently (Midgett *et al.*, 2017), we hypothesized that bile salts directly adhere to ToxR and shield it from protease activity, thereby interfering with ToxR degradation. Midgett *et al*. also indicated that the binding of bile acids facilitates the ToxRS interaction, transducing bile acid signalling into ToxR transcription activity. To address this issue, we monitored PhoA activity of strains with chromosomal *ompT*::*phoA* and *ompU*::*phoA* fusions and ToxR levels in the corresponding strains. We observed that the addition of DC activated *ompU* and repressed *ompT* transcription in WT cells but did not alter the ToxR protein levels in the WT or *toxS* mutant strains (Fig. 6A and B). DC‐dependent activation of ToxR transcriptional activity was significantly decreased in the *toxS* background, supporting the view of Midgett *et al*. (2017) that ToxRS‐bile acids are the relevant activator complex for ToxR. To answer the question of whether the disulfide bond formation or protein stabilization correlates with the DC activation of the ToxR transcriptional activity, the *toxR^CC^* mutant was monitored for *ompU* and *ompT* transcription. The results showed that the *toxR^CC^* strain with the DC treatment could still be transcriptionally activated and showed enhanced ToxR^CC^ protein expression, indicating increased protein stability (Fig. 6A and B). As expected, the *toxR* mutant control showed no activated *ompU* transcription or *ompT* repression with or without DC. To summarize, these results suggest that DC but not TC mediates the protection of ToxR degradation under conditions in which the DegS protease is active. Moreover, we can conclude that ToxS is required for the activation of bile acid‐dependent ToxR transcriptional activity and this does not correlate with a change in the ToxR protein levels.

## Discussion

**Figure 7:**
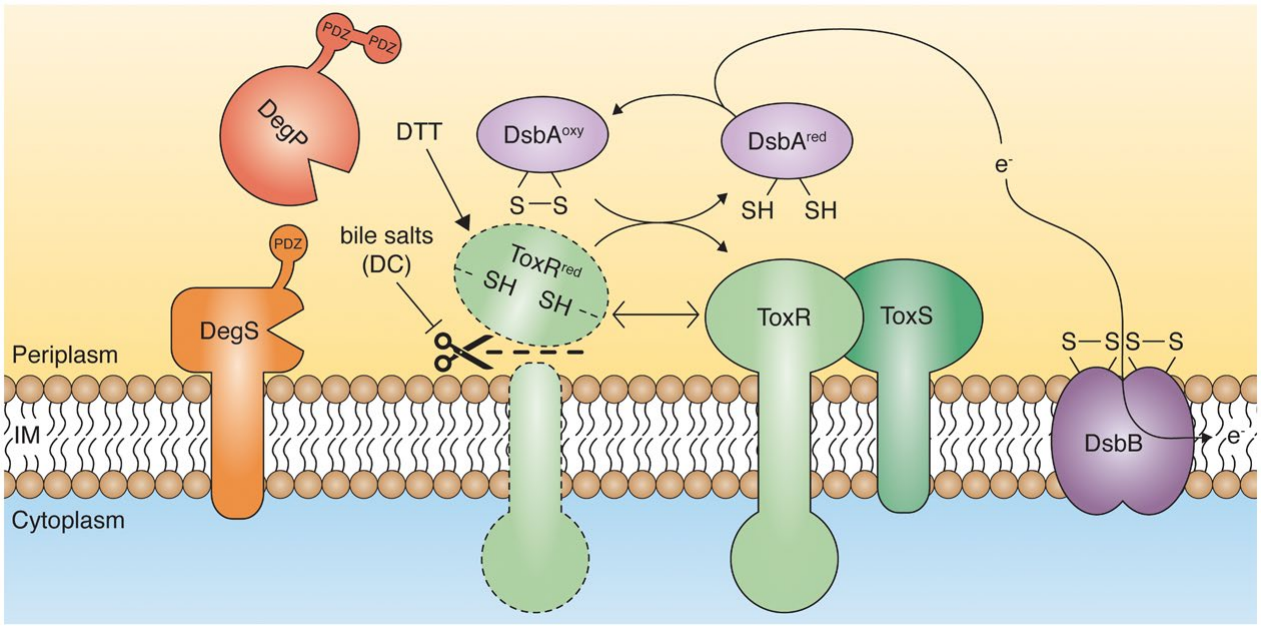
Proteolysis of ToxR is controlled by cysteine‐thiol redox state and bile salts. During de novo synthesis, ToxR molecules are inserted into the inner membrane, exposing thiol groups on the periplasmic located domain. Such thiol groups are then oxidized by the DsbAB system to form intramolecular disulfide bonds. ToxR^oxy^ together with ToxS represents a robust ToxRS transcription complex. Any modifications to the disulfide bond formation of ToxR (e.g. DTT, loss of DsbA activity or exchange of the cysteine residues to serine) lead to DegS‐ and DegP‐mediated proteolysis. Evidence presented here indicates that ToxR molecules, in complex with ToxS, are protected from proteolysis, even under reducing conditions. Standard growth conditions with physiological relevant amounts of bile salts (DC) favour ToxR stability in a ToxS‐ and DegPS‐independent way. [Colour figure can be viewed at https://wileyonlinelibrary.com]

Recently published data showed that *V. cholerae* cells switch into a persistent state under defined laboratory growth conditions, including an alkaline pH and starvation (Almagro‐Moreno *et al.*, 2015a). Such culture conditions exposed the RIP mechanism of ToxR in a RseP (site‐2 protease)‐dependent manner, and further studies indicated the involvement of numerous site‐1 proteases (Almagro‐Moreno *et al.*, 2015b). Our data confirmed this ToxR proteolysis and extended the current model. For example, our analyses revealed that the reduction of ToxR cysteines targets it for site‐1‐mediated proteolysis by DegS and DegP. The impact of the disulfide bond formation has recently attracted increased attention because of its involvement in bacterial virulence and survival (Landeta *et al.*, 2018). In previous observations (Fengler *et al.*, 2012), we demonstrated that *toxR* mutants lacking cysteine residues and *dsbA* strains exhibited decreased ToxR transcriptional activity. This was best demonstrated by the deactivation and the derepression of *ompU* and *ompT* transcription respectively. However, such a phenotype may also correlate well with ToxR proteolysis, prompting us to further investigate ToxR protein stability. To test the fate of ToxR, WT cells were compared with *dsbA, dsbC, dsbAdegS*, FLAG*toxR^CC^* and FLAG*toxR^CC^degPS *mutant strains over a 32 h time course. For the WT strain, monomeric ToxR^red/oxy^ molecules underwent a shift towards the reduced form during entry into the stationary growth phase, and ToxR remained stable at these late time points. In contrast, strong degradation of ToxR/FLAGToxR^CC^ in the *dsbA* and FLAG*toxR^CC^* strains was visible past the 12 h time point. For FLAGToxR^CC^, no degradation was observed in a *degPS* strain over the entire time course of the experiment. For the *degSdsbA* mutant, a delay in ToxR proteolysis was monitored, indicating the activities of additional protease activities at later time points, e.g. DegP. Based on the results of the *degPS* knockout and complementation studies, it appears that the sensitive ToxR^CC^ form is recognized by both proteases independently (Fig. 7). However, this result does not allow for a detailed interpretation since overexpression of proteases may cause artificial effects. Therefore, we cannot currently make inferences on the importance of synergy, additive or sequential mechanisms by which the two proteases act upon ToxR, although these issues will be addressed in future studies. Furthermore, it could be observed that in a *dsbA* strain, the primary fraction of ToxR became ToxR^red^ prior to proteolysis. Since DsbAB activity depends on the respiratory electron chain activity, a change in ToxR^red/oxy^ may correlate with growth status. Complex scenarios may contribute to this activity, for example: (i) a change in the expression of more abundant periplasmic proteins that are substrates for the DsbAB system; (ii) a higher oxidized state of components of the respiratory chain (e.g. ubiquinones) that could act as a better sink for electrons derived from DsbB; or (iii) the existence of a differentially active DsbAB system, either through changes in expression levels or activity and half‐life time. We did not observe ToxR degradation in the *dsbC* mutant but did observe less pronounced ToxR^red^ levels, indicating some change in ToxR^red ^maintenance, a circumstance that requires future study.

To promote cysteine reduction in ToxR, *V. cholerae* was incubated with sub‐lethal concentrations of DTT, after which reduced ToxR monomers became the major detectable form. The level of ToxR undergoing proteolysis was significantly elevated in a *toxS* mutant, which was DegS‐ and DegP‐dependent. Thus, these data confirm that the proteolysis‐sensitive form is the reduced ToxR molecule, which then becomes a substrate for proteolysis, as outlined in the model (Fig. 7). Furthermore, these results are in agreement with previously published data, showing that ToxS is needed to stabilize ToxR (Almagro‐Moreno *et al.*, 2015b; Midgett *et al.*, 2017). As a possible scenario, we assume that the interaction between ToxR and ToxS during de novo synthesis is weakened if ToxR^red^ is dominant, as proteases may act on ToxR. The following observations would support this hypothesis: ToxR mutants that constantly produced or changed the ToxR^red/oxy^ equilibrium to ToxR^red^, such as *dsbA* and *toxR^CC^*, were degraded instantly. Comparatively, treatment with DTT only resulted in ToxR degradation in the absence of ToxS. Therefore, it may be tempting to speculate that if ToxR^red^ is primarily present during de novo synthesis, ToxR und ToxS may not interact strongly. In contrast, if de novo synthesis in the WT strain is not affected in the presence of reducing conditions such that ToxR/ToxS can interact, challenging ToxR with DTT will lead to the production of ToxR^red^, but it could still interact with ToxS. Thus, ToxR^red^ would remain protected from degradation. Future studies are needed to determine which of these scenarios occurs for de novo ToxRS complex assembly.

We also characterized DegS in greater detail. DegS and RseP are part of the σ^E^ stress‐sensor complex, which exclusively cleaves the RseA substrate in *E. coli* (Lima *et al.*, 2013). To the best of our knowledge, no other DegS substrate is known. In this study, as was previously observed (Almagro‐Moreno *et al.*, 2015a), DegS recognizes ToxR in *V. cholerae*. Thus, we were interested in evaluating DegS activity with respect to RseA in *V. cholerae*. As expected, the RseA homologue in *V. cholerae* was confirmed to be a substrate of DegS. Furthermore, the overproduction of a synthetically designed and secreted C‐terminal OmpU oligopeptide, containing a DegS responsive tripeptide signature (YYF) (Walsh *et al.*, 2003; Chaba *et al.*, 2007), also resulted in the stimulation of ToxR^CC^ degradation. Therefore, DegS has similar activation mechanisms in *V. cholerae* as are observed in *E. coli*, but for RseA and ToxR it recognizes two substrates. It would be interesting to determine if more DegS substrates are present in *V. cholerae,* although none have been identified. Additionally, we identified a transcriptional feedback regulatory mechanism between the σ^E^ response and DegP, showing that a *degS* knockout mutant exhibits decreased *degP* transcription. Thus, a *degS* knockout phenotype is also associated with a decrease in DegP levels. Since both proteases are involved in the RIP of ToxR (Fig. 7), it will be interesting to characterize the interaction between the σ^E^ pathway and the ToxR regulon and its physiological importance. A significant indication of a physiologically relevant association between the two systems came from the study by Almagro‐Moreno and colleagues. They showed that σ^E^ pathway‐deficient mutants *rpoE* or *rseP* exhibit no ToxR RIP after being incubated under alkaline and starvation conditions (Almagro‐Moreno *et al.*, 2015b), which interfered with a dormant survival program (Almagro‐Moreno *et al.*, 2015a). In this study, we characterized two key players of the σ^E^ pathway, DegP and DegS, shown to be involved in the control of ToxR proteolysis. We observed that the redox state of ToxR cysteines regulates its proteolysis. Furthermore, we showed that a double *degPS* knockout does not prevent WT ToxR proteolysis under conditions associated with the initiation of a dormant stage. This may simply indicate the existence of unknown environmental conditions that influence the RIP of ToxR in a DegPS and ToxR redox state‐dependent manner.

Previous studies have indicated that bile acids play a major role in stimulating the transcriptional activity of ToxRS (Provenzano and Klose, 2000) and TcpPH (Yang *et al.*, 2013). Furthermore, Midgett and co‐workers reported an enhanced interaction between ToxR and ToxS due to the binding of chenodeoxycholate or cholate (Midgett *et al.*, 2017). To revisit bile acid‐dependent activation, we characterized two primary substituents of bile salts, the sodium salts TC and DC. Interestingly, we demonstrated that incubation with DC but not TC completely blocked FLAGToxR^CC^ degradation in cells and generally enhanced ToxR‐dependent transcriptional regulation of the porin genes *ompU* and *ompT*. DC‐dependent inhibition of RIP on ToxR was also observed under alkaline and starvation conditions, indicating that DC rescues ToxR from becoming a substrate for RIP independently of the proteases. Additionally, we obtained strong evidence that in the presence of bile salts, proteolysis control is ToxS independent, whereas ToxR transcriptional activity is highly accelerated in the presence of ToxS. For ToxR^CC^, a correlation between increased levels of ToxR^CC^ protein and transcriptional activity of porin expression occurs in the presence of DC. Thus, it seems that bile salts activate ToxR independently of disulfide bond formation and protein stability in the WT strain. These analyses also indicated that without notable changes in the WT ToxR levels, the ToxR transcription factor activity was highly stimulated by DC. Similarly, ToxR activity was linked to a different stimulus. Deletions in RND efflux systems were previously observed to lead to the high expression of the *leuO* gene, which is under the control of ToxR (Bina *et al.*, 2018). The accumulation of secreted substances, such as L‐malate, has been shown to lead to binding of activated ToxR, which could constitute a similar mechanism as observed with bile salts (Midgett *et al.*, 2017). However, based on recently published studies, it has become evident that ToxR acts as a receptor for not only bile salts but also perhaps an array of effectors that have yet to be identified.

In summary, our data demonstrated that cysteine oxidation of ToxR is most likely important for proper protein function and stability. Interchangeable dynamics between reduced versus oxidized forms were observable during the transition from early to late stationary growth phases. Furthermore, we showed that ToxR degradation responds to interventions during the disulfide bond formation in a DegS‐ and DegP‐dependent manner. We therefore propose that an increase in thiol group formation and the disruption of the ToxR and ToxS interaction will subsequently lead to ToxR proteolysis, as outlined in the model (Fig. 7).

Several questions remain: What could influence such a thiol redox state switch? Is there an intrinsic half‐life of stable disulfide bonds for ToxR? A good example of regulated disulfide bond formation comes from studies of TcpP homodimer conformation in *V. cholerae* (Yang *et al.*, 2013). There, it was reported that intramolecular disulfide bonds in TcpP are resolved by the presence of TC to form intermolecular disulfide bonds between two TcpP molecules, leading to a homodimer and a transcriptionally active conformation for the *toxT* promoter. Moreover, DsbA and other oxido‐reductases were reported to exhibit activity after binding to TC (Xue *et al.*, 2016). By monitoring the ToxR thiol redox status, we did not observe evidence of a change in the ratio of ToxR^red/oxy^, neither when cells were incubated in alkaline PBS nor in bile salts. Of note, the bile salt concentrations used in this study are physiologically relevant, as was determined in the small intestine of humans (Stadler *et al.*, 1988). The ToxR response to bile acids leads to an inverse regulation of porin expression, associated with cell survival (Provenzano and Klose, 2000). Under these conditions, ToxR may be stabilized to facilitate proper porin regulation. Furthermore, considering that the activation of TcpP depends on bile salts (Yang *et al.*, 2013), it seems that *V. cholerae* has adapted to different bile acid species accordingly and has integrated that in a complex virulence regulatory network. Based on our data, we suggest specific conditions that may lead to ToxR‐regulated RIP, e.g. participation of the σ^E^ pathway leads to an upregulation of DegP, and probably other proteases, due to periplasmic protein folding stress. Varying environments may change the ToxR^red/oxy^ equilibrium due to changes in growth status. Finally, bile acids, which are present during infection cycles of *V. cholerae* in humans or other vertebrates (Hofmann *et al.*, 2010)*,* unexpectedly interfere with ToxR stability. Based on our hypothesis, all three conditions described above may occur in separate parts in the gut and help determine the genetic program of virulence expression due to ToxR control. This complex interaction requires further characterization. In summary, extending the original finding that ToxR proteolysis is involved in environmental persistence (Almagro‐Moreno *et al.*, 2015a), our finding that bile acid regulates the control of ToxR proteolysis may also bolster the concept of ToxR undergoing RIP during the infection cycle.

## Experimental procedures

### Bacteria, plasmids and growth conditions

All bacterial strains and plasmids used in this study are listed in Table 1. *V. cholerae* P27459‐S, a spontaneous streptomycin‐resistant mutant of the clinical isolate P27459 (O1 El Tor Inaba), was used as the wild‐type (WT) strain (Pearson *et al.*, 1993). The *Escherichia coli* strains XL1‐Blue, AB1157, LE392, DH5α λpir and SM10 λpir were used for genetic manipulations, with the latter strain being used for introducing plasmids into *V. cholerae* by conjugation. Bacteria were grown in Luria‐Bertani (LB) broth or on LB agar plates with aeration at 37°C. *V. cholerae* was typically inoculated to a starting OD_600_ of 0.1 and grown to mid‐log phase (OD_600_ of 0.4‐0.5), late‐log phase (OD_600_ of 0.8‐1) or for the indicated time in M9 maltose minimal medium with aeration at 37°C. For starvation and alkaline pH conditions, cells were grown in LB overnight and were resuspended in either PBS or PBS pH 8.1 buffer adjusted with sodium hydrogen carbonate (0.3%). When required, supplements were used at the following final concentrations: streptomycin (Sm; 100 μg ml^–1^), ampicillin (Ap; 50 or 100 μg ml^–1^), chloramphenicol (Cm; 2, 5 or 100 μg ml^–1^), kanamycin (Km; 50 μg ml^–1^), isopropyl ß‐D‐1‐thiogalactopyranoside (IPTG; 0.05 or 1 mM), L‐arabinose (0.05%), sucrose (10%), sodium‐deoxycholate (DC; 0.01 or 0.1%), sodium‐taurocholate (TC; 0.1%) and dithiothreitol (DTT; 6 mM).

**Table 1:** Strains and plasmids used in this study.

Strains/Plasmids	Descriptions	References
*E. coli* strains		
DH5αλpir	*F* *^‐^* Δ(*lacZYA‐argF*)*U169 recA1 endA1 hsdR17 supE44 thi‐1 gyrA96 relA1* λ::*pir*	Hanahan (1983)
SM10λpir	*thi thr leu tonA lacY supE* *recA*::RPA‐2‐Te::Mu λpirR6K, Km^r^	Miller and Mekalanos, (1988)
XL1‐Blue	*F* ^‐^ *::Tn10 * *proA* *^+^* *B* *^+^* *lac* *^q^* Δ*(* *lacZ* *)M151 * *recA1 endA1 gyrA46* *(Nal* *^r^* *) * *thi hsdR17* *(r* *_K_* *^−^* *m* *_K_* *^+^* *) * *supE44 relA1 lac*	Bullock *et al.* (1987)
AB1157	*thr‐1 ara‐14 leuB6 *Δ*(gpt‐proA)62 lacY1 tsx‐33 supE44 * *amber * *galK2 hisG4 rfbD1 mgl‐51 rpsL31 kdgK51 xyl‐5 mtl‐1 argE3 thi‐1*	Bachmann (1987)
LE392	*F* *^‐^* *supF supE hsdR galK trpR metB lacY tonA*	Silhavy *et al.* (1984)
*V. cholerae* strains		
WT	P27459‐S , O1 Inaba, El Tor, clinical isolate, Bangladesh 1976, spontaneous Sm^r^	Pearson *et al.* (1993)
Δ*dsbA*	P27459‐S Δ*dsbA*::*km, dsbA* replaced by *km* cassette, Sm^r^, Km^r^	Fengler *et al.* (2012)
*dsbC*::pGP	P27459‐S *dsbC*::pGP704 insertion, Sm^r^, Ap^r^	Fengler *et al.* (2012)
Δ*toxR*	P27459‐S with deletion in *toxR*, Sm^r^	Fengler *et al.* (2012)
Δ*toxS*	P27459‐S with deletion in *toxS*, Sm^r^	This study
Δ*toxRS*	P27459‐S with deletion in *toxR* and *toxS*, Sm^r^	Fengler *et al.* (2012)
FLAG*toxR* *^CC^*	P27459‐S Δ*toxR*::FLAG*toxR* *^C236SC293S^* *, toxR* replaced by FLAG*toxR* *^C236SC293S^* *, *Sm^r^	Fengler *et al.* (2012)
Δ*toxS* FLAG*toxR* *^CC^*	P27459‐S Δ*toxS* Δ*toxR*::FLAG*toxR* *^CC^* *, toxR* replaced by FLAG*toxR* *^C236SC293S^* *, *Sm^r^	This study
Δ*degS*	P27459‐S Δ*degS*::*cat, degS* replaced by *cat *cassette, Sm^r^ *, *Cm^r^	This study
Δ*degS *Δ*dsbA*	P27459‐S Δ*degS *Δ*dsbA*::*km*, *dsbA* replaced by *km *cassette, Sm^r^ *, *Cm^r^, Km^r^	This study
Δ*toxRS *Δ*degS*	P27459‐S Δ*toxRS *Δ*degS*::*cat,* deletion in *degS* replaced by *cat *cassette, Sm^r^ *, *Cm^r^	This study
Δ*toxS *Δ*degS*	P27459‐S Δ*toxS *Δ*degS*::*cat,* deletion in *degS* replaced by *cat *cassette, Sm^r^ *, *Cm^r^	This study
Δ*toxS* Δ*degPS*	P27459‐S Δ*toxS* Δ*degPS*::*cat*, deletion in *degPS* replaced by cat cassette, Sm^r^, Cm^r^	This study
FLAG*toxR* *^CC ^*Δ*degPS*	P27459‐S FLAG*toxR* *^CC^* Δ*degPS*::*cat, *deletion in *degPS *replaced by *cat* cassette*, *Sm^r^, Cm^r^	This study
WT *ompU*::*phoA*	Insertion of pGP704phoA downstream of *ompU* in P27459‐S, Sm^r^, Ap^r^	This study
Δ*toxS* *ompU*::*phoA*	P27459‐S Δ*toxS* with insertion of pGP704phoA downstream of *ompU*, Sm^r^, Ap^r^	This study
FLAG*toxR* *^CC ^* *ompU*::*phoA*	P27459‐S FLAG*toxR* *^CC^* with insertion of pGP704phoA downstream of *ompU*, Sm^r^, Ap^r^	This study
Δ*toxR ompU*::*phoA*	P27459‐S Δ*toxR* with insertion of pGP704phoA downstream of *ompU*, Sm^r^, Ap^r^	This study
WT *ompT*::*phoA*	Insertion of pGP704phoA downstream of *ompT* in P27459‐S, Sm^r^, Ap^r^	This study
Δ*toxS* *ompT*::*phoA*	P27459‐S Δ*toxS* with insertion of pGP704phoA downstream of *ompT*, Sm^r^, Ap^r^	This study
FLAG*toxR* *^CC ^* *ompT*::*phoA*	P27459‐S FLAG*toxR* *^CC^* with insertion of pGP704phoA downstream of *ompU*, Sm^r^, Ap^r^	This study
Δ*toxR ompT*::*phoA*	P27459‐S Δ*toxR* with insertion of pGP704phoA downstream of *ompT*, Sm^r^, Ap^r^	This study
WT *degP*::*phoA*	Insertion of pGP704phoA downstream of *degP*, Sm^r^, Ap^r^	This study
Δ*degS degP*::*phoA*	P27459‐S Δ*degS *with insertion of pGP704phoA downstream of *degP*, Sm^r^, Ap^r^	This study
Plasmids		
pKEK229	Ori_R6K_, *mobRP4*, *sacB*, Ap^r^	Correa *et al.* (2000)
pCVD442	Ori_R6K_, *mobRP4*, *sacB*, Ap^r^	Donnenberg and Kaper (1991)
pGP704	Ori_R6K_, *mobRP4*, Ap^r^	Miller and Mekalanos (1988)
pBAD18‐Kan	Expression vector, ori_ColE1_, arabinose Inducible, Km^r^	Guzman *et al.* (1995)
pMMB67EH	Expression vector, ori_ColE1_, IPTG inducible, Ap^r^	Morales *et al.* (1991)
pACYC184	Cloning vector, orip15A, Tet^r^, Cm^r^	Rose (1988)
pFLAG‐MAC^TM^	Expression vector with N‐terminal FLAG‐Tag, IPTG inducible, Ap^r^	Sigma‐Aldrich
pTrc99A	Expression vector ori_ColE1_, *lacI* *^q^*, IPTG inducible, Ap^r^	Amann *et al.* (1988)
pKEK229degS::cat	pKEK229 carrying up and down fragments of *degS* flanking a *cat* cassette, Ap^r^, Cm^r^	This study
pKEK229dsbA::km	pKEK229 carrying up and down fragments of *dsbA* flanking a *km* cassette, Ap^r^, Km^r^	Fengler *et al.* (2012)
pCVD442degPS::cat	pCVD442 carrying up and down fragment of *degP* and *degS* flanking a *cat* cassette, Ap^r^, Cm^r^	This study
pCVD442toxS	pCVD442 carrying up and down fragments of *toxS*, Ap^r^	This study
pCVD442toxRS	pCVD442 carrying up fragment of *toxR* and down fragment of *toxS*, Ap^r^	Fengler *et al.* (2012)
pCVD442FLAGtoxR^CC^	pCVD442 carrying up and down fragments of FLAG*toxR* *^C236SC293S^*, Ap^r^	Fengler *et al.* (2012)
pGP704phoA	pGP704 with promoterless *phoA* of SM10λpir, Ap^r^	Berg *et al.* (2007)
pGP704phoAdegP	pGP704phoA with *degP* gene fragment, Ap^r^	This study
pGP704phoAompU	pGP704phoA with *ompU* gene fragment, Ap^r^	This study
pGP704phoAompT	pGP704phoA with *ompT* gene fragment, Ap^r^	This study
pBAD18‐KandegS	*degS* of P27495‐S in pBAD18‐Kan, Km^r^	This study
pMMB67EHdegP	*degP* of P27495‐S in pMMB67EH, Ap^r^	This study
pFLAGrseABC	*rseABC* of P27495‐S in pFLAG‐MAC^TM^, Ap^r^	This study
pFLAGtoxRS	*toxR* and *toxS* of P27495‐S in pFLAG‐MAC^TM^, Ap^r^	Fengler *et al.* (2012)
pFLAGtoxR^CC^toxS	*toxR* *^C236SC293S^* point mutant and *toxS* of P27495‐S in pFLAG‐MAC^TM^, Ap^r^	Fengler *et al.* (2012)
pTrc99ApelB^YYF^	C‐terminal 50 residues of OmpU (ending in YYF) fused to a N‐terminal *pelB* leader sequence in pTrc99A, Ap^r^	This study

### Strain and plasmid constructions

Start‐to‐stop deletions of *toxS*, *degS* and *degPS* in *V. cholerae* P27459‐S were generated via PCR or SOE‐PCR (splicing by overlap extension) (Horton *et al.*, 1989), cloning two approximate DNA fragments (300‐1,000 bp) upstream and downstream of the region of interest into the suicide plasmids pKEK229 or pCVD442 (Donnenberg and Kaper, 1991). To improve the selection of *degS* or *degPS *deletion mutants, a *cat* cassette was obtained from pACYC184 and ligated between the *degS* or the *degPS* upstream and downstream DNA fragments. Corresponding primers for the amplification of the *degPS‐cat*, *degS‐cat *and *toxS* fragments, as well as sequencing primers for validation are listed in Table 2. Synthesized oligonucleotides (Thermo Fisher Scientific) harbouring recognition sites for restriction endonucleases were used to facilitate directed cloning. The resulting constructs were electroporated into *E. coli* DH5α λpir, isolated, transformed into SM10 λpir and subsequently conjugated into *V. cholerae* derivatives (Donnenberg and Kaper, 1991). Double‐crossover recombinant deletion mutants were selected using 10% sucrose agar plates. The *ompU*::*phoA*, *ompT*::*phoA* and *degP::phoA* transcriptional fusion strains were constructed by cloning internal fragments (500‐800 bp) of the respective gene into the plasmid pGP704phoA (Berg *et al.*, 2007), which contains a promoterless *phoA* reporter. After the gene of interest was PCR amplified from the template DNA derived from the WT strain SP27459 and was digested with the corresponding restriction endonucleases, the fragments were ligated into similarly digested pGP704phoA. The resulting constructs were introduced into *V. cholerae* derivatives by conjugation (Miller and Mekalanos, 1988) and were selected for ampicillin resistance.

**Table 2:** Oligonucleotides^a^ (5′‐3′) used in this study.

	
XbaI_dtoxS_fwd		TTTTCTAGATGGATTATTCTAAGTCTGCAT
SacI_dtoxS _rev		ATTGAGCTCCCATGAAACTATTTTTTGTCTC
SOE_dtoxS_rev		TCAGTCAGGAGCAAGATCCTACTCACACACTTTGAT
SOE_dtoxS_fwd		GATCTTGCTCCTGACTGAGCGTAGAATAGGACATAA
HindIII_toxR^CC^_dtoxS_rev		TTTAAGCTTAGCAAGATCCTACTC**AGA**CA
HindIII_dtoxS_fwd		TATAAGCTTCCTGACTGAGCGTAGAATA
NcoI_cat_fwd		TTTACCGGTCAACCAGGCGTTTAAGGG
NcoI_cat_rev		TTTACCGGTTTCCAACTTTCACCATAATGA
SacI_ddegS _fwd		AAAGAGCTCAAAAATCCCCAAACTTGCA
NcoI_ddegS_rev		TAAACCGGTTATAAATATTGACGACGGCA
NcoI_ddegS_fwd		TTTACCGGTCGCCAGAATGTCACAGATA
XbaI_ddegS_rev		ATATCTAGAGAAGCTGGCTAAGAAGTAATGCT
SacI_ompU_fwd		TTTGAGCTCCTTTAATAGTCTATCGAGTTCTT
SthI_ompU_rev		TTTTGGTACCGAAGTACCTTTCGCGCT
SacI_ompT_fwd		ATGAGCTCGGTGATTTGGCTGTGCACG
SthI_ompT_rev		TAGGTACCTTACCAGTAGATACGAGCACC
XhoI_rseA_fwd		TAACTCGAGACTATGGTGAATAGAATGGC
KpnI_rseC_rev		ATTGGTACCTTTCGTTAGGGTTCGCATCA
template for pelB^YYF^		GCCGACCGCTGCTGCTGGTCTGCTGCTCCTCGCTGCCCAGCCGGCGATGGCCCACCACCACCACCACCACTCAGCAGATAATTTTGCTATCGACGCAACTTACTACTTCAAGCCAAACTTCCGCTCTTACATCTCTTACCAGTTCAATCTGCTAGATTCAGACAAAGTTGGTAAAGTAGCATCAGAAGACGAACTGGCTA
NcoI_tripep_fwd		ATTCCATGGGAAAATACCTGCTGCCGACCGCTGCTGCTGGTCT
Pst_YYF_tripep_rev		AATCTGCAGTTAGAA**GTA**GTAACGTAGACCGA TAGCCAGTTCGTCTTCTGATG
bla_inv_rev		CCGTAAGATGCTTTTCTGTGACTGGT
ompU_seq_fwd		CCAACAAACATTAAAATCATTTAA
pFLAGMAC_fwd		AACGGTTCTGGCAAATATTC
pTricHis_rev		CTTCTGCGTTCTGATTTAATCTG
rseB_seq_fwd		GACTCGTGATTCGGTGGA
M13rev‐48_fwd		AGCGGATAACAATTTCAC
SphI_degP_fwd		TTAGCATGCAGGTCGTACCACTGGCT
HindIII_degP_rev		AAAAAGCTTAACGTCCGGTTGAAGT
SacI_depP_phoA‐fusion_fwd		TTAGAGCTCCGGTGGCAACGTCGGTAT
KpnI_degP_phoA‐fusion_rev		TTAGGTACCTTAACGAACAACCAAGTAAAGCG
EcoRI_VC0566_compl_fwd		TTTGAATTCTTTGTTGAGGAGCTTATGATG
BamHI_VC0566_compl_rev		TATGGATCCTTAACGAACAACCAAGTAAAGC
BamHI_degS_fwd		TTTGGATCCTCGCCAGAATGTCACAGAT
SmaI_degS_rev		TTTCCCGGGTGACTGACGAACAGGAAC
SacI_degS_fwd		ATAGAGCTCAAAAATCCCCAAACTTGC
XbaI_degS_rev		ATATCTAGAAGCTGGCTAAGAAGTAAT
HindIII_cat_fwd		ATAAAGCTT AGCACCTCAAAAACACCATC
BamHI_cat_rev		TTAGGATCC CACCAGGCGTTTAAGGGCA
M13_pUC_rev		AGCGGATAACAATTTCACACAGG
pGP704_CVD_rv|15		GATGTAACGCACTGAGAAG

a Restriction sites are underlined

The expression plasmids pBAD18‐KandegS, pMMB67EHdegP, pFLAGrseABC and pTrc99ApelB^YYF^ were constructed by PCR of the respective coding regions derived from the template DNA of the WT strain SP27459. The obtained amplicons were digested with the corresponding restriction endonucleases indicated in the oligonucleotide name (Table 2). For pTrc99ApelB^YYF^, a synthetic oligonucleotide (template for pelB^YYF^, Table 2) comprised of the *pelB* leader sequence and the 3′ region of *ompU* (C’‐terminal 50 amino acid residues) was synthesized (IDT‐Integrated DNA Technologies) and served as template DNA. For the amplification of this fragment, the oligonucleotide primers NcoI_tripep_fwd and Pst_YYF_tripep_rev were used, with the latter containing a point mutation within the DNA sequence that changed the amino acid sequence YDF to YYF for optimized DegS PDZ recognition according to Walsh *et al*. (2003). PCR fragments were ligated into similarly digested pBAD18‐Kan, pMMB67EH, pFLAG‐MAC^TM^ and pTrc99A expression plasmids, which harbour arabinose or IPTG inducible promoters. Subsequently, the expression plasmids were electroporated into DH5α λpir and monitored for kanamycin or ampicillin resistance before being introduced into *V. cholerae* derivatives. All deletion mutants, reporter strains and plasmids described in this study were confirmed by PCR and sequencing (data not shown).

### Cell extracts and immunoblot analysis

WCLs were prepared from *V. cholerae* cultures grown at 37°C in M9 maltose minimal medium (supplemented +/– DTT or bile salts) or incubated under starvation and alkaline conditions (PBS and PBS pH 8.1, respectively) as described above. For time course experiments, cells were harvested at the indicated time points and resuspended in Laemmli buffer (Laemmli, 1970) with or without β‐mercaptoethanol, corresponding to reducing and nonreducing conditions respectively. The protein content was analysed by standard sodium dodecyl sulfate polyacrylamide gel electrophoresis (SDS‐PAGE) using 15% polyacrylamide gels (Mini‐PROTEAN® Tetra cell, BIO‐RAD) with PageRuler™ Prestained Protein Ladder (10‐180 kDa, Thermo Fisher Scientific) as a molecular mass standard. Prior to the immunoblot analysis, WCLs were normalized to contain similar protein levels by SDS‐PAGE Kang staining (Kang *et al.*, 2002).

Immunoblot analysis was conducted using an Amersham^TM^ Protran^TM^ 0.45‐µm nitrocellulose membrane (GE Healthcare Life Sciences). After transfer and blocking in Tris‐Buffered Saline (TBS) with 10% skim milk overnight at 4°C, the membranes were incubated for 2 h at RT with the primary antibody (1:1,000 dilution of rabbit anti‐ToxR (Fan *et al.*, 2014), kindly supplied by Jun Zhu, University of Pennsylvania, USA, or 1:1,000 dilution of mouse anti‐FLAG, Sigma‐Aldrich) diluted in TBS with 10% skim milk. After incubation, the membranes were washed as previously described (Fengler *et al.*, 2012) and then incubated for 1 h at RT with the secondary antibody (1:10,000 or 1:7,500 dilution of horseradish peroxidase‐conjugated goat anti‐rabbit or goat anti‐mouse, Dianova GmbH) diluted in TBS with 10% skim milk. Subsequently, the nitrocellulose membranes were washed, and chemiluminescence detection was performed. Each membrane was incubated for 5 min in ECL solution (Clarity™ Western ECL Blotting Substrates, BIO‐RAD) prior to visualization of the reactive protein bands using a Molecular Imager ChemiDoc^TM^ XRS System (BIO‐RAD). Band intensities were determined using Image Lab Software (BIO‐RAD). Quantification of ToxR protein bands was performed for each strain as follows: for the 0 min time point, the mean densitomertic count numbers of equally treated WCL samples were set to 100% for each strain and conditions (+/– DTT). WCLs were mixed with Laemmli buffer containing ß‐mercapthoethanol such that only the reduced ToxR form was visible and used for quantification. Next, the count numbers for the 30 and 60 min time points were normalized to the 0 min time point on the same immunoblots. Kang‐stained SDS‐PAGE gels served as loading controls to determine equal protein concentrations, similar as shown in Fig. S9 or Fig. S10. One set of immunoblots used for this analysis is shown in Fig. S4.

### Protein stability and degradation assays

To determine ToxR^red/oxy^, FLAGToxR^CC ^and FLAGRseA protein levels in *V. cholerae*, WT strain SP27459 and mutant strains carrying various plasmids (see Table 1) or no plasmid were grown overnight in LB and subcultured into fresh M9 maltose minimal medium to a starting OD_600_ of 0.1 at 37°C. The medium was supplemented with the appropriate antibiotics, and depending on the experiment, with either 0.1% DC or TC (Sigma‐Aldrich). Cultures were grown to mid‐log phase (OD_600_ of 0.4‐0.5), at which point Cm (100 μg ml^–1^) and, if necessary, DTT were added to the bacterial cells to inhibit protein translation, as previously described by Studemann *et al*. (2003). For bacteriaharbouring plasmids, the expression was initially induced by the addition of IPTG (1 mM for pFLAGrseABC and pTrc99ApelB^YYF^ and 0.05 mM for pFLAGtoxR^CC^toxS and pFLAGtoxRS) or arabinose (0.05% pBAD18‐KandegS) for 1 h before protein biosynthesis was blocked by the addition of Cm (100 µg ml^–1^). pMMB67EHdegP was not induced by IPTG since basal expression levels were sufficient to detect DegP activity. Probes without Cm incubation (Cm‐) served as negative controls. WCLs were harvested in Laemmli buffer with or without β‐mercaptoethanol at the indicated time points and analysed by 15% SDS‐PAGE and subsequent immunoblot analysis.

### ToxR transcriptional activity determined by *ompU *and *ompT phoA* fusions

To determine the enzymatic activities for transcriptional *phoA* fusions, alkaline phosphatase activities were assayed as described previously (Miller, 1992). *V. cholerae* strains were grown to late‐log phase (OD_600_ of 0.8‐1) or ON at 37°C in M9 maltose minimal media supplemented with or without DC (0.01%). To note, higher DC concentrations, such as 0.1%, lead to reduced PhoA activity most likely due to a leakiness of the outer membrane, associated with a loss of the PhoA enzyme from the periplasm. Bacterial cells were permeabilized with SDS and chloroform. Experiments were performed in biological triplicates and technical duplicates respectively. The activities were expressed in Miller‐Units: (A_405 _× 1,000)/(A_600 _× ml × min × 0.96).
