# Supplementary material for: Proteolysis of ToxR is controlled by cysteine‐thiol redox state and bile salts in Vibrio cholerae
Source: Mol Microbiol. 2018 Oct 25;110(5):796–810. doi: 10.1111/mmi.14125 (PMC6242745; doi:10.1111/mmi.14125)
Supplement: Supplementary file 1 [file MMI-110-796-s001.pdf]

Supplemental material

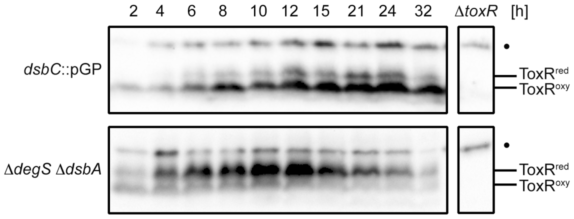

Fig. S1. The cysteine oxidation and reduction state of ToxR affects protein stability. Shown are ToxR immunoblots of *V. cholerae dsbC and degSdsbA* strains grown in M9 maltose. Immunoblots were performed under standard nonreducing Laemmli buffer conditions utilizing α-ToxR antiserum. The migration patterns of ToxR$^{red/oxy}$ are indicated. (•): Represents a nonspecific cross-reacting background band.

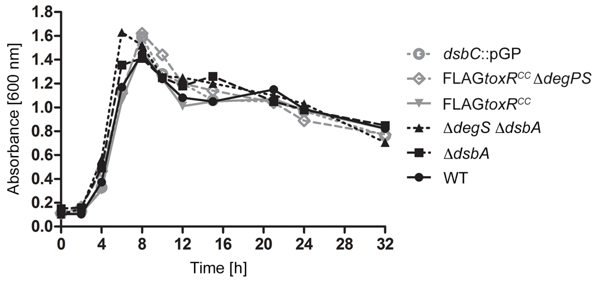

Fig. S2. Growth of *V. cholerae* WT and mutant cells over 32 h. Bacterial cultures were adjusted to an optical density at 600 nm of 0.1 and grown in M9 maltose at 37°C for 32 h. Data are connected to Fig. 1 and Fig. S1.

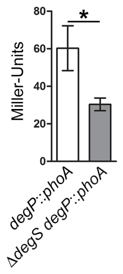

Fig. S3. Transcription of *degP* is affected in a *degS* knockout mutant. Shown are reporter gene activities of alkaline phosphatase PhoA (Miller-Units) linked as operon fusions to *degP* in *V. cholerae*. Strains were grown in M9 maltose ON. Data shown are mean and standard deviation of 6 independent samples. The asterisks indicate a statistically significant difference between WT *degP*::*phoA* and *degS degP*::*phoA* cells, with P-values by paired t-test (*): $P < 0.05$.

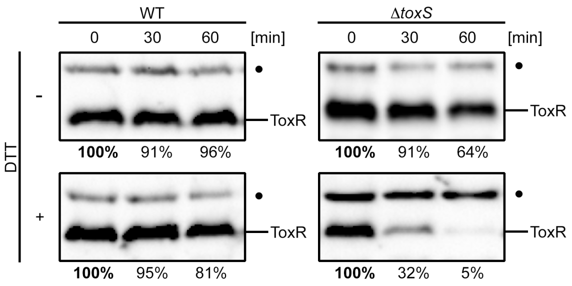

Fig. S4. Effects of DTT treatment on the redox state and protein stability of ToxR in *V. cholerae* WT and *toxS* mutant. Representative immunoblots for densitometric analysis of ToxR temporal stability levels in *V. cholerae* WT and *toxS* strains displayed in Fig. 2B. Strains were grown in M9 maltose with or without DTT (+/-) and time points of sampling are indicated. Protein biosynthesis was inhibited by the addition of Cm. 6 or more of such immunoblots were performed under standard reducing Laemmli buffer conditions utilizing α-ToxR antiserum. ToxR band intensities were readout using Image lab software (BIO-RAD). (•): Represents a nonspecific cross-reacting background band.

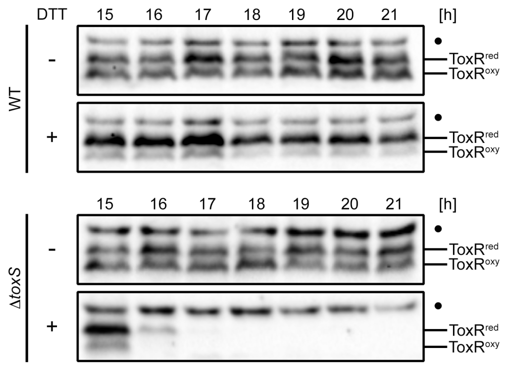

Fig. S5. Effects of DTT treatment on protein stability of ToxR in *V. cholerae* WT and *toxS* mutant. ToxR temporal stability levels were measured by immunoblot analysis in WT and *toxS* mutant strains grown in M9 maltose for 15 h, then incubated for another 6 h with or without DTT (+/-). Immunoblots were performed under standard nonreducing Laemmli buffer conditions utilizing α-ToxR antiserum. The migration patterns of ToxR$^{red/oxy}$ are indicated. (•): Represents a nonspecific cross-reacting background band.

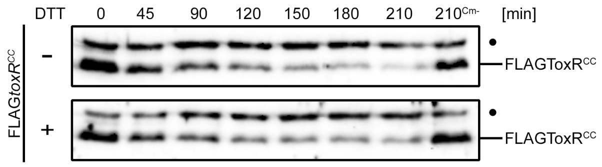

Fig. S6. FLAGToxR^{CC} degradation is not accelerated by DTT. FLAGToxR^{CC} temporal stability levels were measured by immunoblot analysis of *V. cholerae* FLAG*toxR^{CC}* grown in M9 maltose with or without DTT (+/-). Protein biosynthesis was inhibited by the addition of Cm. Samples without chloramphenicol (Cm-) served as negative controls. Immunoblots were performed under standard reducing Laemmli buffer conditions utilizing α-ToxR antiserum. (•): Represents a nonspecific cross-reacting background band.

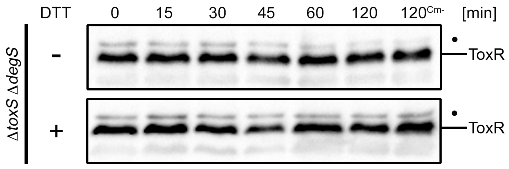

Fig. S7. Deletion of *degS* rescues ToxR^{red} protein in the absence of ToxS. ToxR temporal stability levels were measured by immunoblot analysis in *toxSdegS* mutant strain grown in M9 maltose with or without DTT (+/-). Protein biosynthesis was inhibited by the addition of Cm. Samples without chloramphenicol (Cm-) served as negative controls. Immunoblots were performed under standard nonreducing Laemmli buffer conditions utilizing α-ToxR antiserum. The migration patterns of ToxR$^{red/oxy}$ are indicated. (•): Represents a nonspecific cross-reacting background band.

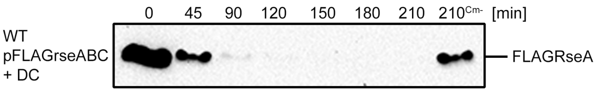

Fig. S8. Sodium deoxycholate (DC) does not inhibit DegS activity. WT cells harbouring pFLAGrseABC were grown in M9 maltose supplemented with 0.1% DC and subsequently induced with IPTG for 1 h. Protein biosynthesis was inhibited by the addition of Cm. Samples without chloramphenicol (Cm-) served as negative controls. Immunoblots were performed under standard reducing Laemmli buffer conditions utilizing anti-FLAG antibodies to monitor temporal expression levels of FLAGRseA.

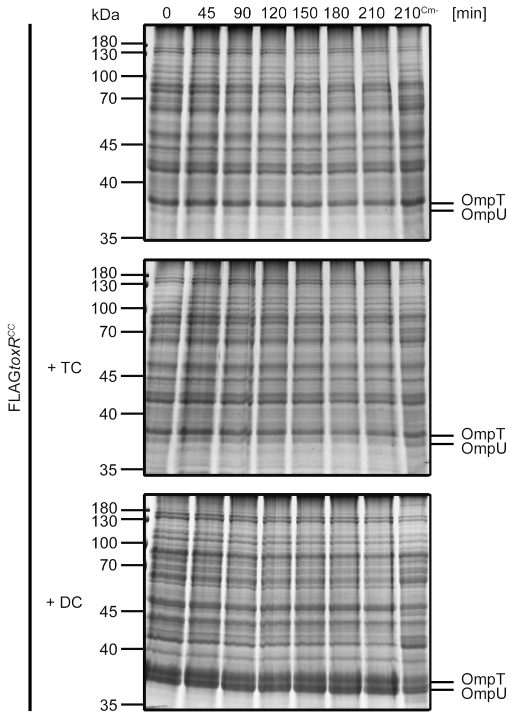

Fig. S9. Comparative analysis of protein amounts and OmpU/T expression patterns derived from bile salt treated cells. WCL of *V. cholerae* FLAG*toxR*^{CC} were analysed by SDS-PAGE and Kang staining as loading controls for immunoblots displayed in Fig. 4. Cells were grown in M9 maltose or media supplemented with 0.1% DC. Protein biosynthesis was inhibited by the addition of Cm and samples without chloramphenicol (Cm-) served as negative controls.

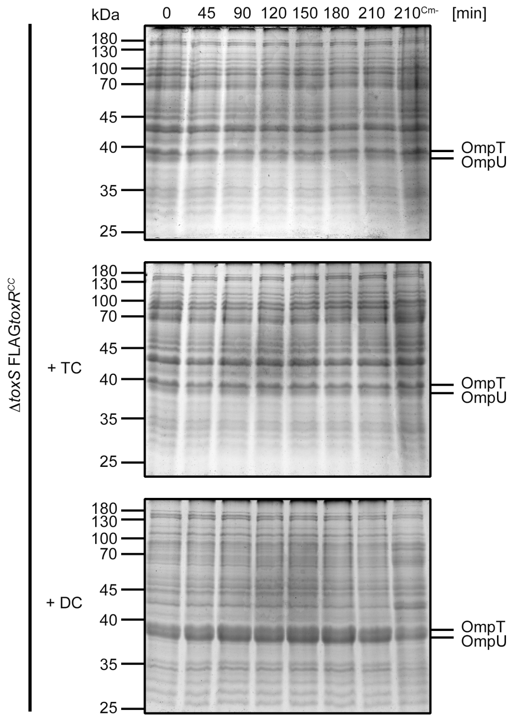

Fig. S10 Comparative analysis of protein amounts and OmpU/T expression patterns derived from bile salt treated cells. WCL of *V. cholerae toxS* FLAG*toxR*^{CC} were analysed by SDS-PAGE and Kang staining as loading controls for immunoblots displayed in Fig. 4. Cells were grown in M9 maltose or media supplemented with 0.1% DC. Protein biosynthesis was inhibited by the addition of Cm and samples without chloramphenicol (Cm-) served as negative controls.
